# Exploring the risk of glycemic variability in non-diabetic depressive individuals: a cross-sectional GlyDep pilot study

**DOI:** 10.3389/fpsyt.2023.1196866

**Published:** 2023-09-15

**Authors:** Shivang Mishra, Anurag Kumar Singh, Sumit Rajotiya, Pratima Singh, Preeti Raj, Hemant Bareth, Mahaveer Singh, Tushar Jagawat, Deepak Nathiya, Balvir Singh Tomar

**Affiliations:** ^1^Department of Pharmacy Practice, Institute of Pharmacy, Nims University Rajasthan, Jaipur, India; ^2^School of Public Health, University of Alberta, Edmonton, AB, Canada; ^3^Department of Endocrinology, National Institute of Medical Sciences, Nims University Rajasthan, Jaipur, India; ^4^Department of Psychiatry, National Institute of Medical Sciences, Nims University Rajasthan, Jaipur, India; ^5^Department of Clinical Studies, Fourth Hospital of Yulin (Xingyuan), Yulin, Shaanxi, China; ^6^Department of Clinical Sciences, Shenmu Hospital, Shenmu, Shaanxi, China; ^7^Institute of Pediatric Gastroenterology and Hepatology, Nims University Rajasthan, Jaipur, India

**Keywords:** depression, glycemic variability, risk of diabetes, FGM, CES-D, glycemic variability indices

## Abstract

**Background:**

Data on the correlation between glycemic variability and depression in nondiabetic patients remain limited. Considering the link between increased glycemic variability and cardiovascular risks, this relationship could be significant in depressed patients.

**Methods:**

In this single-center pilot study, we utilized Flash Glucose Monitoring (Abbott Libre Pro) to study glycemic variability. The CES-D (Center for Epidemiological Studies– Depression) scale was employed to measure depression levels. Based on CES-D scores, patients were classified into two groups: those with scores ≥ 33 and those with scores < 33. We analyzed various glycemic variability indices, including HBGI, CONGA, ADDR, MAGE, MAG, LI, and J-Index, employing the EasyGV version 9.0 software. SPSS (version 28) facilitated the data analysis.

**Results:**

We screened patients with depression visiting the department of psychiatry, FGM was inserted in eligible patients of both the groups which yielded a data of 196 patient-days (98 patient-days for CES-D ≥ 33 and 98 patient-days for CES-D < 33). The glycemic variability indices CONGA (mg/dl), (76.48 ± 11.9 vs. 65.08 ± 7.12) (*p* = 0.048), MAGE (mg/dl) (262.50 ± 25.65 vs. 227.54 ± 17.72) (*p* = 0.012), MODD (mg/dl) (18.59 ± 2.77 vs. 13.14 ± 2.39) (*p* = 0.002), MAG(mg/dl) (92.07 ± 6.24vs. 63.86 ± 9.38) (*p* = <0.001) were found to be significantly higher in the CES-D ≥ 33 group.

**Conclusion:**

Patients with more severe depressive symptoms, as suggested by CES-D ≥ 33, had higher glycemic variability.

## 1. Introduction

Diabetes mellitus, a global health issue, stands as one of the prevalent non-communicable diseases impacting millions worldwide. Beyond the well-researched complications of neuropathy, nephropathy, retinopathy, and cardiovascular sequelae, there emerges a significant shadow of psychological morbidity, most profoundly depression. This complex relationship is substantiated by recent meta-analyses, such as those conducted by Mezuk et al. (1) and Chireh et al. (2), which indicate that diabetes increases the risk of developing depression by approximately 25% (1, 2). The relationship between diabetes and depression is bidirectional. Diabetes can elevate the risk of developing depression, and similarly, depression can predispose one to diabetes. When they coexist in an individual, it’s not just a simple overlap. This confluence exacerbates the progression and complicates the outcomes of both disorders.

Depression, characterized by pervasive mood disturbances, underpins profound implications for metabolic health, particularly glycemic control. A confluence of pathophysiological mechanisms including inflammation, neuroendocrine dysfunction, and alterations in insulin dynamics have been implicated in mediating this association (3). Longitudinal studies further emphasize the chronic impact of depression on glycemic variability (GV), a parameter depicting fluctuations in blood glucose levels that has been linked to microvascular complications and oxidative stress (4).

However, the majority of these studies are conducted in diabetic populations and rely on traditional glucose monitoring systems, which may not accurately capture the day to day spectrum of GV. Recent innovations like the FreeStyle Libre flash glucose monitoring system offer a more nuanced window into these fluctuations, yet there is a paucity of research exploring the depression-GV nexus in non-diabetic individuals using this technology. Observational studies have highlighted the potential connections, but more targeted research is needed (5).

The objective of this research is to fill this research gap through a pilot study examining the relationship between depression severity and GV in non-diabetic individuals, employing the advanced FreeStyle Libre system. This cross-sectional GlyDep Pilot Study seeks to extend the current understanding of this complex interplay by focusing on a population often overlooked in conventional research. By shedding light on the mechanisms at play in non-diabetic individuals, the findings may pave the way for early interventions and personalized therapeutic strategies that account for both mental and metabolic health. By engaging with cutting-edge technology and a novel demographic, this study endeavors to contribute a fresh perspective to the ongoing discourse surrounding depression, GV, and their broader implications for public health (6).

## 2. Methodology

The present study was conducted in compliance with the ethical principles of the Declaration of Helsinki, and approval for the study was obtained from the Institutional Review Board (approval number: NIMSUR/IEC/2022/211). All study subjects provided informed consent for this observational analysis.

### 2.1. Design and participants

The present study, called GlyDep, is a primary quantitative exploratory research project aimed at analyzing glycemic variability (GV) in individuals with depressive disorder. Recruitment of participants, aged 18 years and older, diagnosed with depression (ICD-10) was conducted at the Department of Psychiatry, National Institute of Medical Science and Research in Jaipur, India, from April 2022 to November 2022. Diagnosis of incident depression was based on ICD-10 codes F32 (all mild to severe depressive episodes) or F33 (all recurrent depressive disorders) with cognitive behavioral therapy for management of diabetes (7). The study utilized a set of inclusion and exclusion criteria. Inclusion criteria consisted of a proven diagnosis of depression, glycated hemoglobin indices A1c (HbA1c) levels <5.6%, and willingness to give consent for the study. Patients were excluded if they did not meet the clinical diagnosis according to ICD-10, had unstable severe medical conditions such as active malignant diseases, heart failure, or chronic liver diseases, were below 18 years of age, or had HbA1c levels above 5.6%.

### 2.2. Data collection and recruitment of the study population

#### 2.2.1. Demographic factors

Customized data collection forms were designed and used to collect the study data. Participants’ age, gender, marital status, smoking habits, and educational status were documented in the data collection forms. Weight and height were measured as per protocol and the body mass index (BMI) was calculated. The criteria established by the World Health Organization for overweight (23.0 kg/m2) and obesity (25.0 kg/m2) were used to determine BMI status (8). Body composition was assessed with waist and hip measurements, which was obtained from standard measuring tape. Waist-to-hip ratio (WHR) was calculated by dividing the waist circumference by the hip circumference (9).

#### 2.2.2. Laboratory parameters

The participants lipid profile was assessed, including Low-Density Lipoprotein (LDL), High-Density Lipoprotein (HDL), and Triglycerides (TG), following the guidelines of the American Heart Association (AHA) (10). Additionally, Blood Urea Nitrogen (BUN), Serum Creatinine (SCr), Aspartate Aminotransferase (AST), and Alanine Transaminase (ALT) levels were measured. These particular measurements are integral for monitoring kidney function and liver health, ensuring a comprehensive evaluation of the participants’ overall metabolic health.

#### 2.2.3. Glucose assessment

##### 2.2.3.1. HbA1c measurement

HbA1c serves as a sensitive indicator of long-term glycemic control, reflecting average blood glucose levels over a period of approximately 2 to 3 months. In this study, HbA1c levels were measured *via* high-performance liquid chromatography (HPLC) of hemolysates from whole blood (<5.6%) which is a reliable and gold standard technique for HbA1C determination. Glucose levels in fasting serum samples were assessed using glucose oxidase peroxidase and a Siemens Dimension EXL 200 analyzer.

##### 2.2.3.2. Flash glucose monitoring (Freestyle libre Pro)

In this study, the ambulatory glucose profile was calculated using interstitial sensor glucose data obtained from the Freestyle Libre Pro system (Abbott Diabetes Care, Oxon, UK). The system comprised a sensor worn by patients for 2 weeks, which tested interstitial glucose levels at 15-min intervals (11). All study participants were instructed to wear the sensor for the entire two-week period, resulting in a total of 196 patient-days.

Glycemic variability indices, such as mean sensor glucose and its standard deviation (SD), absolute means of daily differences (MODD), continuous overall net glycemic action (CONGA), mean amplitude of glycemic excursion (MAGE), high blood glucose index (HBGI), mean absolute glucose (MAG), liability index (LI), average daily risk ratio (ADRR), and J-Index were among the glycemic variability indices. EasyGV version 9.0 software (University of Oxford, OX2 6GG, United Kingdom) was utilized to compute the above indices using the data collected for 196 patient-days (Review Supplementary Table 1) (12–17).

#### 2.2.4. Measures for depressive symptoms (CES-D)

The Center for Epidemiological Research Depression Scale (CES-D) was devised by the National Institute of Mental Health in the 1970s. Its primary intent was to assess depressive symptomatology in the general population, bridging the gap between clinical diagnosis and population-based assessment. Over the years, it has been adapted for various subpopulations and has become one of the widely accepted tools for screening depression symptoms in epidemiological studies.

Compared to other depression scales, CES-D uniquely incorporates a range of symptoms, capturing diverse domains such as mood, somatic complaints, and interpersonal interactions. This holistic approach ensures a comprehensive understanding of an individual’s depressive state. The Center for Epidemiological Research Depression Scale (CES-D) was employed to screen for depression symptoms under the guidance of a designated psychiatrist (18) The CES-D contains 20 items commonly used in screening for depression and depressive symptoms. The CES-D response options were based on recent symptoms and a 4-point Likert scale ranging from “rarely or none of the time” to “most or all of the time.” The scale goes from 0 to 60, with a higher score indicating more significant depressive symptoms (19).

Cronbach’s alpha was 0.85 in reliability testing (20). Furthermore, significant correlations with other depression measurement scales were observed, supporting the convergent validity of the CES-D, and construct validity was established by differences between psychiatric inpatients and the general population (19).

### 2.3. Statistical analysis

We used IBM SPSS version 28.0 from Chicago, IL, United States for our statistical analysis. We summarized continuous variables with mean and standard deviation, while categorical variables were presented as frequency and percentage. To compare differences between groups, we used t-tests for continuous variables and Fisher’s exact test for categorical variables. Acknowledging our cautious approach toward our small sample’s uniqueness and potential data non-normality, we found non-parametric statistical methods to be necessary. Since finding non-diabetic participants posed challenges, we explored alternative methods. Non-parametric tests, known for their reliability with limited data, became suitable choices. We emphasize awareness of assumptions and limitations in both parametric and non-parametric analyses. Furthermore, Microsoft Excel 2015 facilitated data visualization.

## 3. Results

### 3.1. Study population

**Figure 1:**
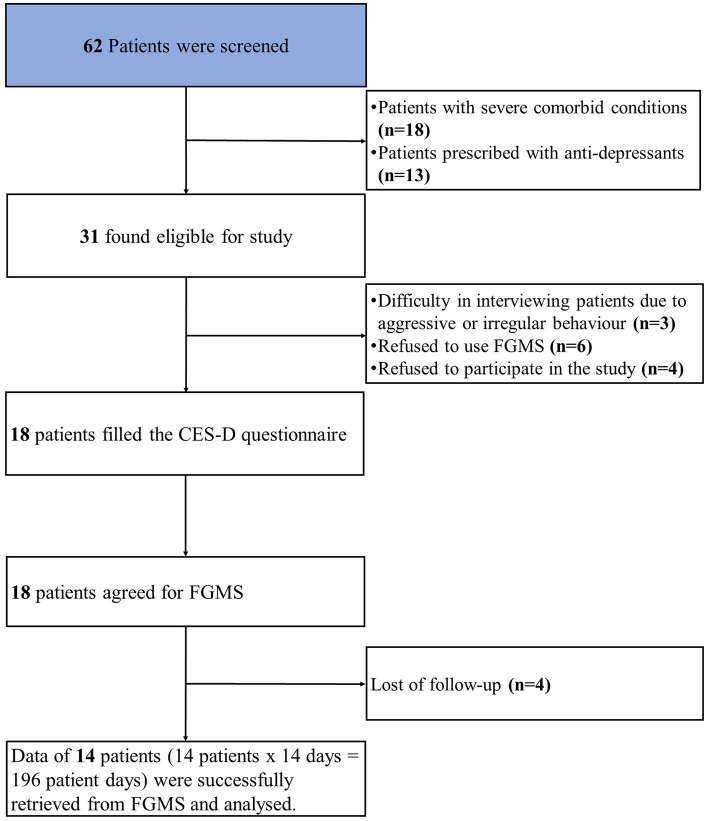
Study flow chart showing enrollment and exclusion of the study subjects.

At the psychiatry outpatient department of NIMS hospital, we screened 62 patients for our study. Out of 62 patients with depression, thirty-one patients were found to be eligible for the study. Out of thirty-one, thirteen patients were excluded from the study. The reasons for the exclusion were as follows: (1) difficulty in interviewing patients due to aggressive or irregular behavior (*n* = 3); (2) refusal to use FGMS (*n* = 6); (3) refusal to participate in the study (*n* = 4). Finally, eighteen patients were enrolled, with a loss of follow-up (*n* = 4). The study flow chart is shown in Figure 1.

The study included 14 participants with a total of 196 patient-days. Of 14 participants 10 were males and 4 were females with an average age of 29.53 ± 1.77 years. We made two groups depicting the severity of depression: CES-D scores≥33 (6 males, 1 female) and < 33 (4 males, 3 females). The overall CES-D score was 33.46 ± 7.32 (range: 0–60), 39.71 ± 3.81 for the CES-D ≥ 33 group, and 27.00 ± 2.70 for the CES-D < 33 group. The comparison of the data of patients who had CES-D > 33 to those who had CES-D < 33 is shown in Table 1. Age and HbA1C were significantly higher in the patients with CES-D ≥ 33 (Table 1).

**Table 1:** Baseline comparison of the patients as per the CES-D score, a score used to depict the severity of depression.

Variables	< 33 (*n* = 7)	≥ 33 (*n* = 7)	*p*- value
Age, (years)	24.14 (4.05)	36.42 (4.10)	0.047
Male, *n* (%)	4 (40)	6 (60)	0.559
Married, *n* (%)	4 (40)	6 (60)	0.559
**Education status**			
Primary school, *n* (%)	0 (0)	1 (14.2)	0.510
Intermediate, *n* (%)	5 (71.42)	4 (57.14)	
Graduate or Post graduate, *n* (%)	2 (28.57)	2 (28.57)	
Smokers, *n* (%)	2 (28.57)	4 (57.14)	0.290
BMI, (kg/m^2^)	22.17 (2.56)	23.20 (4.24)	0.594
WHR, mean ± SD	0.91 ± 0.03	0.92 ± 0.03	0.599
HbA1c (%)	4.82 ± 0.59	5.52 ± 0.34	0.020
LDL, (mg/dl), mean ± SD	85.74 ± 21.85	89.42 ± 24.16	0.770
HDL (mg/dl), mean ± SD	45.37 ± 16.41	53.08 ± 14.56	0.371
TG (mg/dl), mean ± SD	143.42 ± 143.76	152.00 ± 72.75	0.890
BUN (mg/dl), mean ± SD	10.82 ± 3.42	10.81 ± 3.38	0.998
SCr (mg/dl), mean ± SD	0.77 ± 0.26	0.85 ± 0.17	0.489
AST (U/L), mean ± SD	42.14 ± 43.73	21.14 ± 7.88	0.235
ALT (U/L), mean ± SD	68.14 ± 71.00	35.14 ± 9.52	0.246

All the data is presented in mean ± standard deviation (SD) or number (*n*) and percentage (%).

BMI, Body Mass Index; WHR, Waist Hip Ratio; HbA1c, Glycated Hemoglobin; LDL, Low-Density Lipoprotein; HDL, High-Density Lipoprotein; TG, Triglycerides; BUN, Blood Urea Nitrogen; SCr, Serum Creatinine; AST, Aspartate aminotransferase; ALT, Alanine transaminase; CES-D, Center for Epidemiologic Studies– Depression.

### 3.2. Distribution of glycemic variability indices

**Figure 2:**
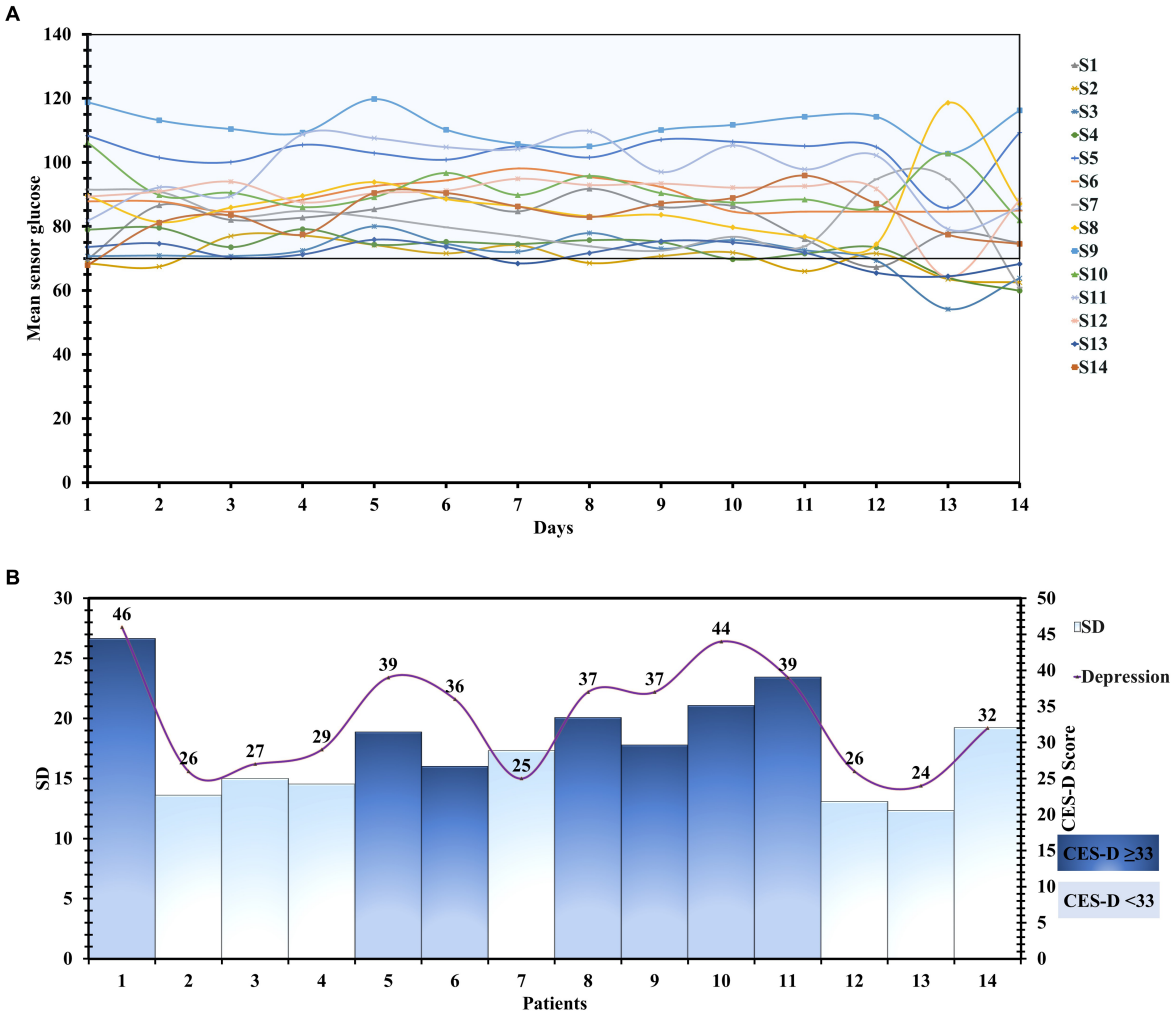
**(A)** Detailed day-wise tracing of the sensor glucose values of 196 patient-days. **(B)** The Mean glucose level and Standard deviation of all the patients with their CES-D scores.

Supplementary Table 2 shows an explanatory version of the measures of glycemic variability, along with their mean and standard deviations. The standard deviation of the blood glucose, a marker of glycemic variability, was higher in CES-D ≥ 33 group (Figures 2A,B).

### 3.3. Glycemic variability and depression

We compared the glycemic variability indices of the patients who had CES-D ≥ 33 to those who had CES-D < 33. The HbA1c was higher in the patients who had CES-D ≥ 33 (5.52 ± 0.34 vs. 4.82 ± 0.59) (*p* = 0.020) (Table 1).

**Figure 3:**
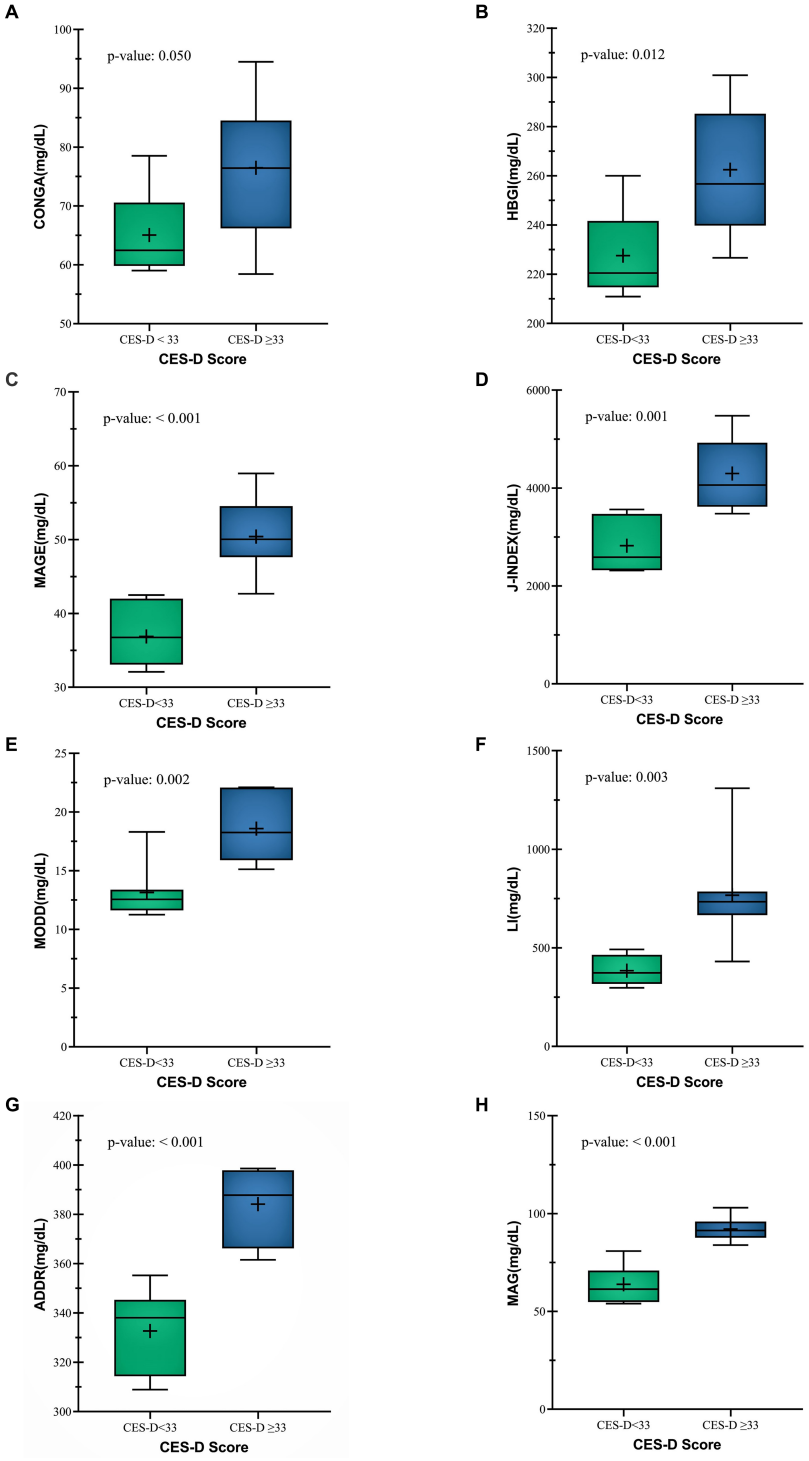
Comparison between Glycemic variability indices **(A)** CONGA **(B)** HBGI **(C)** MAGE **(D)** J-INDEX **(E)** MODD **(F)** LI **(G)** ADDR **(H)** MAG of both CES-D groups.

CONGA (mg/dl) was higher in CES-D ≥ 33 group (76.48 ± 11.9 mg/dL vs. 65.08 ± 7.12 mg/dL) (*p* = 0.048) (Figure 3A). Likewise, HBGI (mg/dl) and MAGE (mg/dl) values were also higher (50.41 ± 5.21 vs. 36.89 ± 4.09) (*p* = <0.001), (262.50 ± 25.65 vs. 227.54 ± 17.72) (*p* = 0.012) respectively (Figures 3B,C) Other glycemic variability indices like J-Index (mg/dl) (4296.49 ± 777.98 vs. 2822.79 ± 526.53) (*p* = 0.001), MODD (mg/dl) (18.59 ± 2.77 vs. 13.14 ± 2.39) (*p* = 0.002), LI(mg/dl) (766.74 ± 266.28vs. 384.41 ± 72.98) (*p* = 0.003), ADDR (mg/dl) (384.14 ± 15.43 vs. 332.71 ± 17.21) (*p* = <0.001) and MAG(mg/dl) (92.07 ± 6.24vs. 63.86 ± 9.38) (*p* = <0.001) were also found to be significantly higher in the CES-D ≥ 33 group (Figures 3D–H). These findings show that glycemic variability was higher in patients with a CES-D score ≥ 33.

## 4. Discussion

This research endeavors to fill the void of understanding concerning glycemic variability in non-diabetic patients with depression. To assess the patients’ glycemic variability, the FGMS was utilized for a period of 2 weeks, which generated an ambulatory glucose profile of 196 patient-days. The glycemic indices were calculated *via* the utilization of EasyGV version 9.0 software. Depression was assessed using the CES-D scale, which has been validated in the Indian population. Patients were assigned to two groups based on their CES-D scores, with scores <33 and scores ≥33. The results of this study reveal that patients with CES-D scores ≥33 exhibited increased glycemic variability.

The etiology of elevated glycemic variability in individuals with depression is multifactorial. In depression, there is an upsurge in stress hormones, particularly cortisol, which can be severe enough to result in pseudo- cushing syndrome (21). The elevated cortisol acts on the subcortical area, including the hippocampus and hypothalamus (22). These two areas are crucial for the control of the autonomic nervous system regulation. Autonomic dysfunction, as observed in patients with diabetes, has been linked to elevated glycemic variability. This has been seen in patients with diabetes, who have autonomic dysfunction, and had high glycemic variability (23, 24). The glycemic variability was also found to be associated with incident depression. In a retrospective study from the Korean National Health Insurance Service–National Health Screening Cohort from 2002 to 2007, patients (n-264,480) who have at least three fasting serum glucose were later observed during 2008–2013 (n-198,267), and their hazard ratios (HR) of incident depression were calculated. After adjustment, it was found that the highest glycemic variability was associated with a 9% increased risk of depression (HR, 1.09; 95% CI, 1.02–1.16). The risk of incident depression heightened with increasing GV (*p* for trend < 0.001) (22). In our pilot study, we tried to explore the glycemic variability in depressive patients. Our pilot study had the objective of exploring glycemic variability among non-diabetic individuals with depression. The heightened glycemic variability observed in patients with CES-D scores ≥33 suggests an elevation in stress hormone levels.

Additionally, there exists a connection between glycemic variability and endothelial dysfunction, which is a precursor to atherosclerosis and cardiovascular incidents. Notably, depression itself is also linked to endothelial dysfunction. The coexistence of both conditions may potentially contribute to an increased risk of cardiovascular events.

In summary, our pilot study illuminates the correlation between glycemic variability and depression in individuals without diabetes. The noted rise in stress hormones among those exhibiting higher CES-D scores highlights the importance of this link. Moreover, the interaction among glycemic variability, endothelial dysfunction, and depression underscores potential repercussions for cardiovascular well-being (25).

### 4.1. Future recommendations

In this study, our objective is to underscore patient education and awareness initiatives that highlight the link between glycemic variability and depression. Advocating for holistic care includes integrating comprehensive management strategies and interdisciplinary consultations. Expanding this research to a larger, diverse cohort is imperative to bolster the association regarding glycemic variability, particularly in non-diabetic populations. Our recommendation is to enhance robust methodologies by controlling confounders and predictors, encompassing dietary habits, physical activity, medication usage, and lifestyle factors. Embracing these approaches propels progress in patient care and scientific understanding, ultimately enhancing overall well-being.

### 4.2. Limitations

This pilot research represents a pioneering application of a flash glucose monitoring system to evaluate glycemic variability among patients afflicted with depression, who do not suffer from diabetes. Moreover, the glycemic variability is analyzed relative to the severity of the depression. Nevertheless, certain constraints were observed during the study. The principal restriction was the restricted sample size, which may limit the generalizability of the findings. Additionally, the low screening-to-enrollment ratio was attributed to the social stigma surrounding depression in India, which also served as a significant contributing factor to the attrition of study participants.

## 5. Conclusion

Patients who have more severe depression (CES-D scores≥33) have high glycemic variability (SD, MAGE, CONGA, and MODD) than the patients who have less severe depression (CES < 33).

## Data availability statement

The data collected and/or evaluated in this study are intended for academic research and can be accessed upon suitable request to the corresponding authors.

## Ethics statement

The study was conducted in accordance with the Declaration of Helsinki, and approved by the Institutional Ethics Committee, NIMS University Rajasthan, Jaipur (IEC Approval number: NIMSUR/IEC/2022/211) on 26 March 2022 for studies involving humans. The participants provided their written informed consent to participate in this study.

## Author contributions

MS: conceptualization, investigation, validation, and writing—original draft. SM: conceptualization, investigation, validation, and writing—original draft. PS: methodology and project administration. DN: conceptualization, validation, resources, writing—original draft, supervision, and funding. SR and AS: investigation, validation, and writing—original draft. PR: investigation, formal analysis, and writing—original draft. HB: investigation, formal analysis, writing, reviewing, and editing. TJ: investigation, writing, reviewing, and editing. BT: conceptualization, resources, writing, reviewing, editing, supervision, and funding. All authors contributed to the article and approved the submitted version.
