# Supplementary material for: Exploring the risk of glycemic variability in non-diabetic depressive individuals: a cross-sectional GlyDep pilot study
Source: Front Psychiatry. 2023 Sep 15;14:1196866. doi: 10.3389/fpsyt.2023.1196866 (PMC10541025; doi:10.3389/fpsyt.2023.1196866)
Supplement: Supplementary file 1 [file Table_1.docx]

**Supplementary Table 1: Description for various Glycemic variability indices.**

| **Sr. No.** | **Glycemic Variability Indices** | **Definitions** |
| --- | --- | --- |
| **1.** | M-VALUE | Is a weighted average of the glucose values, with progressively larger penalties for more extreme values: M-VALUE = \|10 × log10 (Gn/IGV) \|3, where IGV is the ideal glucose value assumed equal to 120 mg/dl (12). |
| **2.** | LBGI (Low Blood Glucose Index) | Is a transformation that normalizes the blood glucose scale: LBGI = 1.509 × {[loge (Gn)]1.084 – 5.381}, for blood glucose values <112.5 mg/dl (13). |
| **3.** | High Blood Glucose Index (HBGI) | 1. Is a transformation to normalize the blood glucose scale, for blood glucose values higher than 112.5 mg/dl (13). |
| **4.** | Average Daily Risk Range (ADRR) | Is the sum of LBGI and HBGI, calculated with the minimum and the maximum glucose value, respectively (14). |
| **5.** | J-INDEX | 1. Is a combination of information from GMEAN and GSD: J-INDEX = 0.001 × (GMEAN+GSD)2 (15). |
| **6.** | CONGA (Continuous Overlapping Net Glycemic Action) | 1. Is the standard deviation (SD) of the difference between values typically obtained 60 min apart (16). |
| **7.** | MAGE (Mean Amplitude of Glycemic Excursion) | Is the mean of the glycemic excursions that are >1 SD (17). |
