# Supplementary material for: Exploring the risk of glycemic variability in non-diabetic depressive individuals: a cross-sectional GlyDep pilot study
Source: Front Psychiatry. 2023 Sep 15;14:1196866. doi: 10.3389/fpsyt.2023.1196866 (PMC10541025; doi:10.3389/fpsyt.2023.1196866)
Supplement: Supplementary file 2 [file Table_2.docx]

| **CES-D** | **≥ 33** | | | | | | | **< 33** | | | | | | | **Total Average** |
| --- | --- | --- | --- | --- | --- | --- | --- | --- | --- | --- | --- | --- | --- | --- | --- |
| **Measure** | **P1** | **P5** | **P6** | **P8** | **P9** | **P10** | **P11** | **P2** | **P3** | **P4** | **P7** | **P12** | **P13** | **P14** |  |
| **MEAN** | 83.44 ± 6.68 | 103.68 ± 2.41 | 91.69 ± 4.48 | 83.95 ± 5.72 | 111.27 ± 4.13 | 89.97 ± 3.48 | 101.74 ± 6.74 | 71.83 ± 3.6 | 73.6 ± 3.22 | 74.72 ± 2.88 | 79.54 ± 5.88 | 91.97 ± 1.99 | 72.13 ± 3.2 | 86.48 ± 5.07 | 80.68 ± 16.19 |
| **SD** | 24.76 ± 4.06 | 18.59 ± 1.89 | 15.1 ± 2.98 | 17.95 ± 4.38 | 17.05 ± 3.45 | 19.32 ± 4.28 | 20.35 ± 3.85 | 12.15 ± 1.76 | 14.74 ± 1.81 | 12.46 ± 2.17 | 13.71 ± 3.65 | 12.22 ± 1.94 | 12.01 ± 1.84 | 16.2 ± 5.28 | 17.78 ± 4.12 |
| **CONGA** | 60.71 ± 5.24 | 85.07 ± 3.52 | 78.67 ± 5.65 | 67.21 ± 5.85 | 93.65 ± 5.16 | 74.63 ± 3.06 | 82.69 ± 6.08 | 59.38 ± 3.86 | 60.14 ± 3.29 | 63.91 ± 3.04 | 67.54 ± 6.82 | 78.79 ± 1.77 | 60.28 ± 2.7 | 73.35 ± 5.99 | 70.78 ± 11.13 |
| **LI** | 1468.9 ± 530.95 | 743.95 ± 159.62 | 381.78± 194.82 | 720.65 ± 371.1 | 737.82 ± 402.64 | 768.93 ± 373.8 | 847.84 ± 347.43 | 278.17 ± 68.99 | 387.67 ± 120.75 | 328.64 ± 161.67 | 396.65 ± 194.11 | 319.48 ± 130.08 | 291.28 ± 104.08 | 482.37 ± 552.99 | 575.57 ± 273.02 |
| **JINDEX** | 3820.10 ± 622.77 | 4846.82 ± 202.77 | 3702.53 ± 326.4 | 3383.4 ± 544.38 | 5346.85 ± 506.72 | 3883.45 ± 485.63 | 4854.32 ± 687.96 | 2290.06 ± 202.36 | 2533.67 ± 227.71 | 2467.57 ± 226.71 | 2828 ± 362.69 | 3521.07 ± 221.94 | 2299.76 ± 231.08 | 3432.54 ± 504.93 | 3559.64 ± 996 |
| **HBGI** | 237.70 ± 15.55 | 283.75 ± 5.02 | 259.14 ± 10.01 | 240.96 ± 13.06 | 299.04 ± 7.79 | 254.59 ± 7.21 | 279.18 ± 13.66 | 213.41 ± 9.23 | 217.27 ± 7.86 | 220.62 ± 6.96 | 231.67 ± 14.14 | 260.48 ± 4.22 | 214.25 ± 7.93 | 247.21 ± 11.44 | 245.02 ± 27.88 |
| **MAGE** | 65.72 ± 14.64 | 46.59 ± 5.42 | 39.14 ± 8.03 | 51.61 ± 14.72 | 48.4 ± 13.87 | 56.54 ± 22.83 | 59.02 ± 13.51 | 30.97 ± 5.38 | 36.51 ± 5.3 | 35.72 ± 10.97 | 36.51 ± 7.99 | 32.65 ± 5.67 | 33.27 ± 5.95 | 36.6 ± 13.52 | 43.65 ± 8.33 |
| **MVALUE** | 1307.05 ± 124.82 | 1684.86 ± 43.76 | 1469.74 ± 83.15 | 1323.15 ± 106.34 | 1820.09 ± 71.68 | 1434.62 ± 62.56 | 1647.48 ± 120.55 | 1097.17 ± 70.14 | 1129.57 ± 61.67 | 1152.43 ± 54.89 | 1243.01 ± 111.85 | 1476.99 ± 36.43 | 1102.92 ± 61.65 | 1371.78 ± 94.37 | 1360.67 ± 233.22 |
| **MAG** | 101.27 ± 10.33 | 93.73 ± 10.71 | 82.95 ± 8.79 | 99.99 ± 18.11 | 85.48 ± 11.48 | 94.54 ± 14.01 | 77.95 ± 16.48 | 65.77 ± 7.17 | 64.76 ± 10.74 | 51.36 ± 6.53 | 77.7 ± 20.65 | 52.39 ± 8.45 | 60.91 ± 12.53 | 69.16 ± 6.71 | 77.96 ± 16.51 |
| **MEAN BG: Mean blood glucose, SD: Standard deviation, CONGA: Continuous overall net glycemic action, LI: Liability index, HBGI: High blood glucose index, MAGE: Mean average glycemic excursion, MAG: Mean absolute glucose**  **#P (1-14): Patients (1-14)**  ***All the data shown is in (Mean** **± SD) format** | | | | | | | | | | | | | | | |

**Supplementary Table 2: Detailed Glycemic variability indices of all the enrolled patients.**
